# Phylogeographic Characterization of Tick-Borne Encephalitis Virus from Patients, Rodents and Ticks in Slovenia

**DOI:** 10.1371/journal.pone.0048420

**Published:** 2012-11-20

**Authors:** Luka Fajs, Emina Durmiši, Nataša Knap, Franc Strle, Tatjana Avšič-Županc

**Affiliations:** 1 Institute of Microbiology and Immunology, Faculty of Medicine, University of Ljubljana, Ljubljana, Slovenia; 2 Department of Infectious Diseases, University Medical Center Ljubljana, Ljubljana, Slovenia; University of Minnesota, United States of America

## Abstract

Tick-borne encephalitis virus (TBEV) is the most important arboviral agent causing infections of the central nervous system in central Europe. Previous studies have shown that TBEV exhibits pronounced genetic variability, which is often correlated to the geographical origin of TBEV. Genetic variability of TBEV has previously been studied predominantly in rodents and ticks, while information about the variability in patients is scarce. In order to understand the molecular relationships of TBEV between natural hosts, vectors and humans, as well as correlation between phylogenetic and geographical clustering, sequences of TBEV E and NS5 protein genes, were obtained by direct sequencing of RT-PCR products from TBE-confirmed patients as well as from rodents and ticks collected from TBE-endemic regions in Slovenia. A total of 27 partial E protein gene sequences representing 15 human, 4 rodent and 8 tick samples and 30 partial NS5 protein gene sequences representing 17 human, 5 rodent and 8 tick samples were obtained. The complete genome sequence of TBEV strain Ljubljana I was simultaneously obtained. Phylogenetic analysis of the E and NS5 protein gene sequences revealed a high degree of TBEV variability in patients, ticks and rodents. Furthermore, an evident correlation between geographical and phylogenetic clustering was shown that was independent of the TBEV host. Moreover, we show the presence of a possible recombination event in the TBEV genome obtained from a patient sample, which was supported with multiple recombination event detection methods. This is the first study that simultaneously analyzed the genetic relationships of directly sequenced TBEV samples from patients, ticks and rodents and provides the largest set of patient-derived TBEV sequences up to date. In addition, we have confirmed the geographical clustering of TBEV sequences in Slovenia and have provided evidence of a possible recombination event in the TBEV genome, obtained from a patient.

## Introduction

Tick-borne encephalitis virus (TBEV) is the most important arboviral agent in central Europe, responsible for thousands of cases of central nervous system infections every year. TBEV is a member of the family *Flaviviridae*, genus *Flavivirus* [1]. TBEV genome comprises a single-stranded positive-sense RNA of approximately 11 kb, which encodes a polyprotein that is cleaved into three structural and seven non-structural proteins [2]. Phylogenetic analyses of TBEV isolates identified three TBEV subtypes: European (TBEV-Eu), Far Eastern (TBEV-Fe) and Siberian (TBEV-Sib) [3]. TBEV is transmitted by *Ixodes* spp. ticks. Primary vector of the TBEV-Eu subtype strains is *Ixodes ricinus*. TBE is maintained in the ecosystem between tick and vertebrate hosts. The most important vertebrate reservoirs in Europe are rodents. Rodents serve as both maintaining and amplifying hosts [1]. Although TBEV viral load is often low or undetectable, they play an important role in the circulation of the virus in nature as they enable co-feeding of infected and uninfected ticks [4].

In TBE-endemic areas the virus is distributed patchily in geographically limited areas, known as TBE-foci [1], [5]. Within such foci, several genetic variants of TBEV have been identified [6]–[12]. Furthermore, several studies have identified geographical clustering of specific TBEV variants within small geographic areas [6]–[8]. Gaumann et al. for example performed phylogenetic analysis of 71 TBEV found in *I. ricinus* in Switzerland and identified 10 phylogenetic clades of closely related TBEV, some of which showed regional clustering [6]. Furthermore, Weidmann et al. have also identified the presence of several viral variants in ticks in Czech Republic and Germany. Their analysis also showed regional clustering of TBEV variants and a correlation between the genetic and geographic distances of TBEV isolates. They were thereby able to determine the direction of spread of TBEV in Central Europe in a westerly direction [7]. These studies were focused on determination of genetic variability of TBEV in rodents and ticks, but little is known about genetic variability of TBEV in patients in endemic areas.

While the incidence of disease has been shown to be correlated with the regional prevalence of TBEV in ticks and rodents [8], [13], [14] limited information is available on the relatedness of TBEV strains among different hosts in endemic areas. Durmiši et al. [8] have shown for example that TBEV is genetically related in ticks and patients in Slovenia. The aim of our study was to expand the knowledge about the genetic relatedness of TBEV in rodents, ticks and patients in Slovenia. Furthermore, we aim to determine the overall genetic variability of TBEV in Slovenia and assess if there is a correlation between phylogenetic and geographic clustering of TBEV variants in Slovenia in all three hosts. While previous studies were focused on molecular determination of virus isolates, direct sequencing of RT-PCR products was employed in our study in order to avoid the occurrence of genetic variation due to virus culturing or cloning. Thereby we were able to obtain a more realistic view of the genetic variability of TBEV in Slovenia. We report the largest number of patient-derived TBEV sequences to date, and show that geographical clustering of TBEV variants is correlated to the phylogenetic clustering and is independent of the host. We also provide evidence of a possible recombination event in the TBEV genome obtained from a patient.

## Results

**Figure 1:**
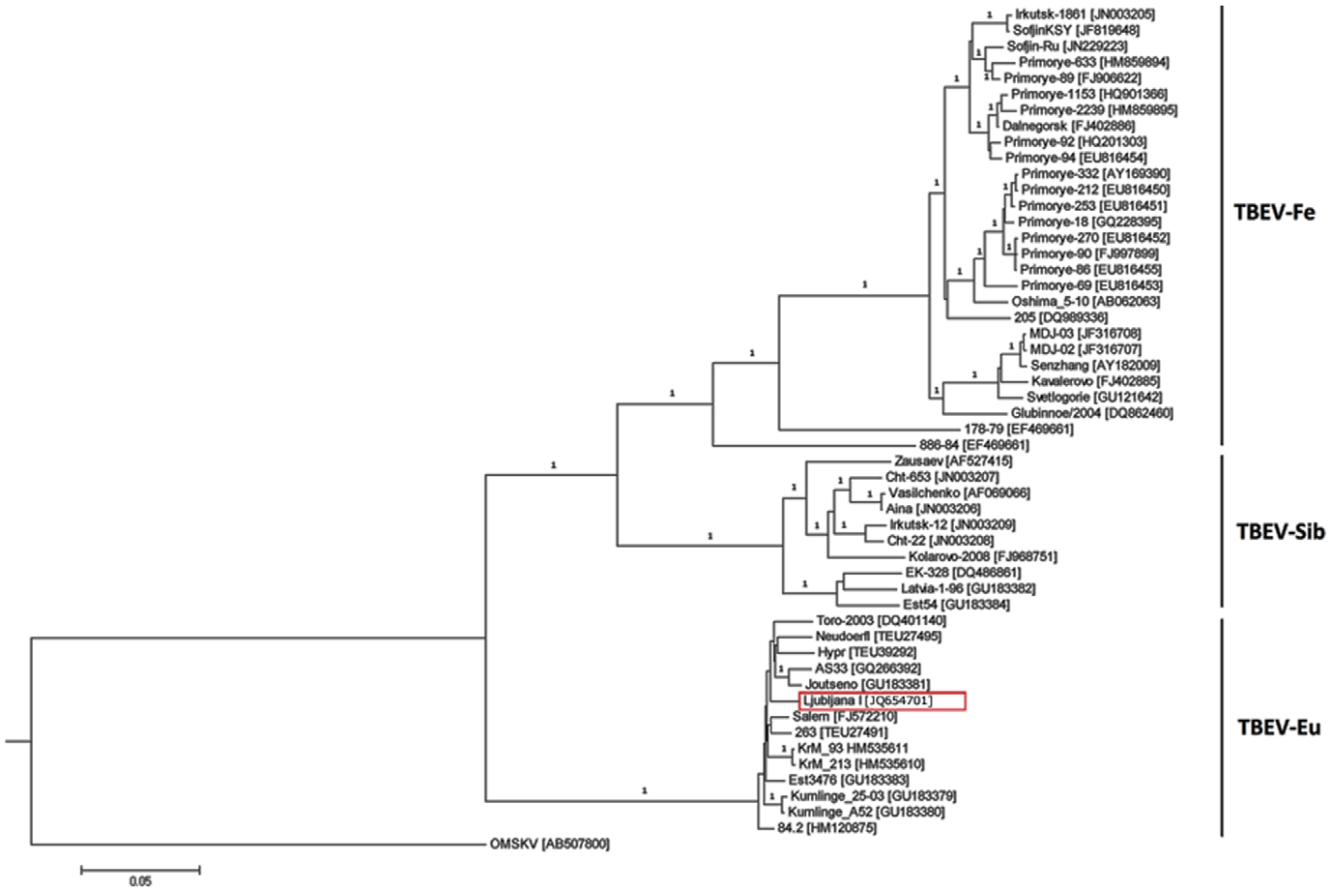
Bayesian phylogenetic analysis of the complete genome sequence of TBEV strain Ljubljana I. Branch labels represent posterior probabilities. GenBank accession numbers of all sequences are shown in the brackets alongside TBEV strain names. OMSKV = Omsk hemorrhagic fever virus.

Complete genome sequence of TBEV strain Ljubljana I was determined and deposited in the GenBank database under accession number JQ654701. Genome organization analysis of the 11,092 bp long sequence revealed that it was identical to that of other members of the TBEV group. Phylogenetic analysis revealed that strain TBEV Ljubljana I belongs to the TBE-Eu subtype (Figure 1) and is most closely related to the Central European TBEV strains Salem and 263 both on the nucleotide level and the deduced amino acid level (divergence 2.0% and 0.6%). When TBEV strain Ljubljana I complete genome sequence was compared with 51 complete genomes of TBEV deposited in GenBank, a total of 7 unique amino acid substitutions were detected.

A total of 27 partial E protein gene sequences were obtained by direct sequencing of RT-PCR products from clinical samples of TBE-confirmed patients, ticks and rodents: 15 from human samples, 8 from tick samples and 4 from rodent samples. A 757 bp long segment of the E protein gene was used for phylogenetic analysis. Nucleotide sequence identity ranged from 95.6 to 100% (divergence 0–4.4%) whereas the identity on the amino acid level ranged from 98.4–100% (divergence 0–1.6%). The most prominent amino acid change compared to the TBE-Eu reference strain Neudörfl was I167V, which was present in all Slovenian sequences. In addition, a larger part of the E protein gene (1164 bp) sequence was obtained from 20 samples. A total of 15 amino acid changes were detected compared to the reference Neudörfl strain. The most prominent amino acid changes compared to the Neudörfl strain were the same as observed in the 757 bp long segment. Phylogenetic analysis of the 1164 bp segment revealed that the 757 bp long segment was equally informative and therefore the 757 bp segment was used for further analyses.

A total of 30 partial NS5 protein gene sequences were obtained: 17 from human samples, 8 from tick samples and 5 from rodent samples. A 933 bp long segment of the NS5 protein gene was used for phylogenetic analysis. The identity of the determined TBEV NS5 gene nucleotide sequences ranged from 96.7 to 100% (divergence 0–3.3%) whereas the identity on the amino acid level ranged from 97.4–100% (divergence 0–2.6%). A total of 8 amino acid changes were detected compared to the Neudörfl strain. The most prominent amino acid changes were V51M and K108R which were present in all Slovenian sequences.

**Figure 2:**
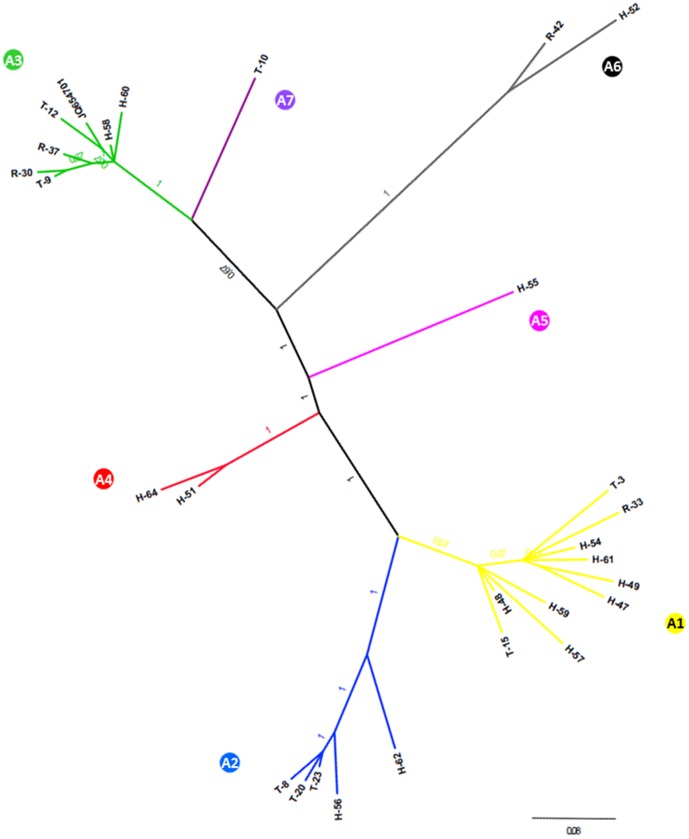
Bayesian phylogenetic analysis of the partial E protein gene sequences (757 bp) from human, tick and rodent samples in Slovenia. Branch labels represent posterior probabilities. Sample information is given in Table 1. Designations A1–A7 represent the determined phylogenetic clusters.

**Figure 3:**
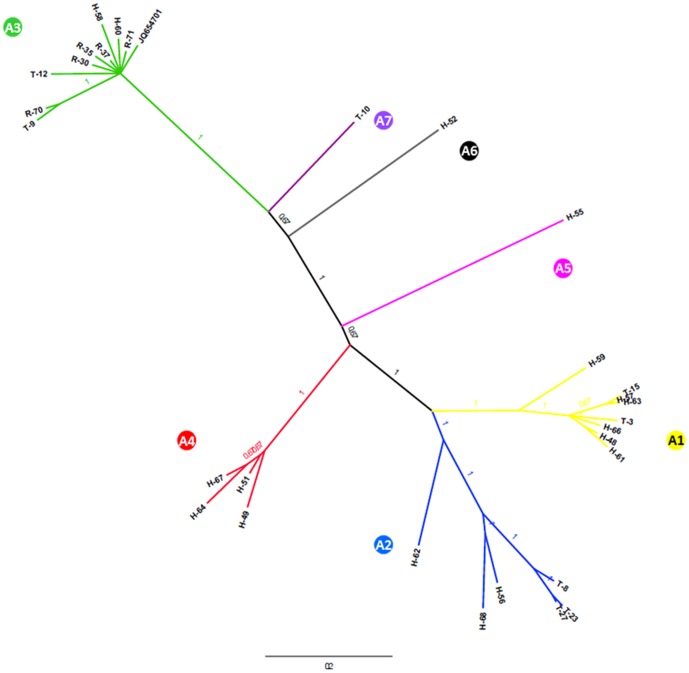
Bayesian phylogenetic analysis of the partial NS5 protein gene sequences (933 bp) from human, tick and rodent samples in Slovenia. Branch labels represent posterior probabilities. Sample information is given in Table 1. Designations A1–A7 represent the determined phylogenetic clusters.

Phylogenetic analysis of the 757 bp TBEV E gene sequences revealed that sequences grouped in three major clades (A1–A3) and four additional minor clades (A4–A7), with high posterior probabilities (Figure 2). Phylogenetic clustering was independent of the TBEV host. Namely, TBEV sequences from ticks, rodents and patients clustered together. Similar results were obtained from the phylogenetic analysis of the 933 bp long TBEV NS5 protein gene sequences (Figure 3). Three major and four minor clades were formed (A1–A7). Sequences that clustered together in the E protein gene phylogenetic analysis also clustered together in the NS5 protein gene phylogenetic analysis. The only major exception was clustering of a TBEV sequence obtained from a patient sample H-49. While it clustered within clade A1 based on the E protein gene phylogenetic analysis, it clustered within clade A4 based on the NS5 protein gene phylogenetic analysis. Because the resulting branch swap could had occurred due to a recombination event, we addressed this question by analyzing concatenated E and NS5 protein gene sequences from samples where both were available (n = 22). Analysis revealed a putative recombination event in the E protein gene region of sample H-49 including two parental sequences H-51 (cluster A5) and H-61 (cluster A1). The recombination event was most strongly supported by the 3Seq algorithm (average p-value 9.108×10−4) and less with algorithms MaxChi (4.623×10−2), Chimaera (3.404×10−2) and SiScan (1.129×10−2).

**Figure 4:**
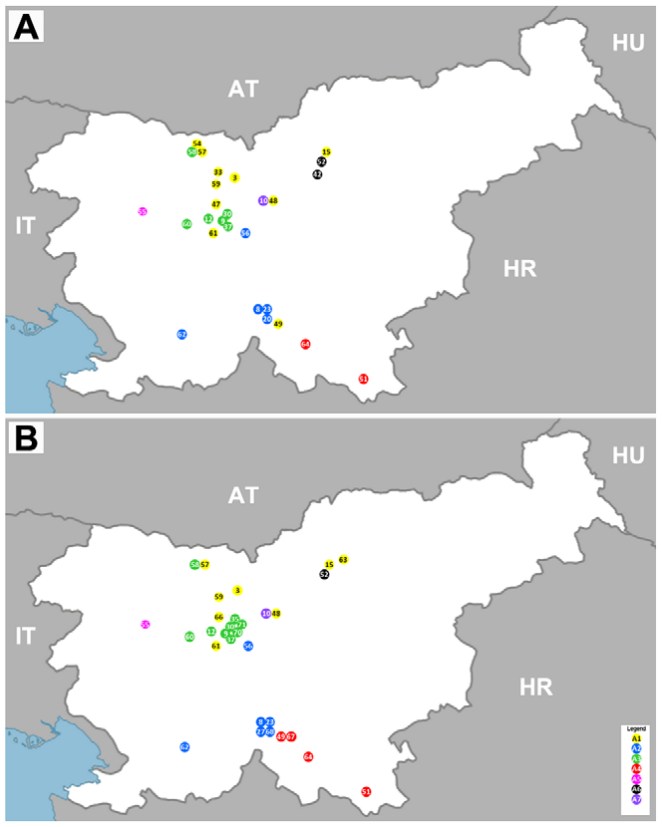
Correlation of geographical and phylogenetic clustering based on A) TBEV E sequences and B) TBEV NS5 sequences. Figure shows the geographic distribution of sample isolation sites of tick and rodent sample or location of patients' residency with respect to the phylogenetic clustering of their partial A) TBEV E and B) NS5 protein gene sequences. Legend shows the coloring of isolates from different clades; Clade A1: yellow placemark, clade A2: blue placemark, clade A3: green placemark, clade A4: red placemark, clade A5: pink placemark, clade A6: black placemark, clade A7: violet placemark. Numbers in the placemarks represent sample ID.

To assess potential correlation of sequence divergence to geographical locations, sample isolation locations were plotted on the map of Slovenia and colored according to their respective phylogenetic clustering based on E and NS5 protein gene sequence analysis (Figure 4). As shown in Figure 4, a high level of regional clustering was present both after E and NS5 protein gene sequence analysis. Sequences from clades A1 and A3 were obtained from samples in the northern and central part of Slovenia while the sequences from clades A2 and A4 were obtained mostly from the southern part of Slovenia. Some level of regional clustering was observed also within clades A1, A3 and A2, A4: sequences from clade A1 being present even more to the north of Slovenia than the majority of sequences in clade A3 and sequences from clade A2 being present in the south-western part of Slovenia and sequences from clade A4 being present in the south-eastern part of Slovenia. As expected, the largest discordance was observed for the putatively recombinant sequence from sample H-49. Interestingly, no correlation was observed between genetic and geographic distances despite the evident correlation between geographical and phylogenetic clustering. Therefore, direction of spread of TBEV in Slovenia could not be determined.

Furthermore, no correlation was observed between the years of sampling and phylogenetic clustering.

## Discussion

The aim of our study was to determine the genetic diversity of TBEV virus variants present in TBE endemic regions in Slovenia from human, tick and rodent samples. Nucleotide sequences were obtained by directly sequencing RT-PCR products from these samples in an attempt to avoid the occurrence of mutations that could arise in the process of virus culturing or cloning. To the best of our knowledge this is the first study that employed direct determination of nucleotide sequences and simultaneous analysis of TBEV sequences from three TBEV hosts in one geographical area. We have shown that sequences from all three analyzed TBEV hosts cluster together. This observation is in concordance with the low mutation rate of TBEV and its conservative nature due to selective pressures implicated by the course of host switching. These results also imply that a limited number of genetic changes occur in the course of human infection. With respect to the quick course of disease and the low mutation rate of TBEV, these results are expected.

Relatively high genetic variability of Slovenian TBEV was observed. Seven phylogenetic clades were determined both on the E and NS5 protein gene sequence analyses. Previous studies of genetic diversity of TBEV have also shown that multiple sequence variants are present in relatively small geographical areas. Genetic variability of the Slovenian TBEV variants is similar to those reported in previous studies (nucleotide sequence identity 96.1 to 100%) [6], [7], [10], [15], [16]. Our results also revealed that TBEV variants in Slovenia show correlation between geographical and phylogenetic clustering which is in concordance with previous reports [8]. Clustering was most evident for sequences in clades A1 and A3, which were obtained in the northern part of Slovenia and sequences in clades A2 and A4 which were present predominantly in the southern part of Slovenia. The observed distribution could be attributed to natural (levels of forestation) and human (freeways and larger cities) factors that prevent migration of hosts from the northern to the southern part of Slovenia and vice versa. In a recent study, Weidmann et al. [7] analyzed TBEV sequences from ticks in the Czech Republic and Germany, and have shown that TBEV was spread in the Central Europe from the east to west. Since in our study no correlation was observed between genetic and geographical distances we could not assess the direction of spread of TBEV in Slovenia. Namely, sequences from geographically related clades did not exhibit higher genetic identities. This was most profoundly seen in the analysis of clades A1 and A3, which show greatest genetic divergence but originate from geographically close locations. These results could indicate that TBEV was introduced in Slovenia on multiple occasions.

In order to confirm these findings a larger number of TBEV sequences from the eastern and western part of Slovenia should be included. Correlation between phylogenetic and geographical clustering was evident also on a smaller scale. Namely, ticks and rodents from the same or nearby isolation sites shared a high level of sequence identity, whereas ticks and rodent from different locations showed greater sequence divergence. A similar but not as evident trend was also seen in human samples. Of note, the phylogeographic analysis of the human samples included the locations of residency which do not necessarily correlate to the site of infection. In Slovenia nationwide travel is frequent due to the short distances and therefore it is not unlikely that patients with residence in one part of Slovenia got infected in other parts of Slovenia. On the other hand, despite the frequent migrations a geographically and phylogenetically well separated cluster of four patients in the south-eastern part of Slovenia was observed (clade A4), implying that this variant most likely also circulates in rodents and ticks in that area.

Of note are also minor clades A5–A7 that were supported by the analysis of both E and NS5 protein gene sequences, which reveal an even greater genetic diversity of TBEV in Slovenia. In order to confirm this observation, further sampling of ticks and rodents in that area would be necessary.

Analysis also revealed, that the phylogenetic clustering was not correlated to the year of sampling, therefore a temporal effect of the observed phylogenetic clustering was excluded.

An interesting finding was the observed differential clustering of TBEV E and NS5 protein gene sequences obtained from a patient sample H-49. E protein gene phylogenetic analysis revealed clustering in clade A1, while the NS5 protein gene phylogenetic analysis showed clustering in clade A4. Because, such phylogenetic jumps often arise due to recombination events, we tested this hypothesis with several recombination event detection methods. Analyses confirmed the possible recombination event between a virus from clade A4 and a virus from clade A1. Because rigorous contamination control measures were undertaken during this study, we believe that these results are most reliable. Reports on recombination in tick-borne viruses are scarce. To best our knowledge the only reported possible recombination event were detected by Uzcategui et al. [17] and Yun et al. [18]. Although no major recombination events were detected in these studies, several minor recombination events were discovered. In this view, our study gives the first evidence of possible recombination within different TBEV variants from patients. Because these results are preliminary in nature, further sampling of ticks and rodents in these areas are necessary to gain greater insight into whether the recombination event is a consequence of a dual infection of the patient or the recombinant variant is circulating in Slovenia.

In conclusion, our study provides the largest set of patient-derived TBEV sequences and for the first time compares TBEV sequences from rodents, ticks and patients from one endemic area. All sequences were obtained by direct sequencing of RT-PCR products. Our results show that similar TBEV variants are present in all three hosts. Furthermore, our analysis revealed relatively high genetic variability of TBEV and evidence of geographical clustering of TBEV in Slovenia. In addition, we provide evidence of a possible recombination event in TBEV genome obtained from a patient.

## Materials and Methods

### Samples

TBEV strain Ljubljana I isolated in 1992 from blood of a TBE patient was used for the completion of the whole genome sequence [19]. Low-level passage was used for the determination of the complete genome.

Previously confirmed TBEV RNA-positive samples were selected from a collection of TBE-confirmed patient, tick and rodent samples stored at the Laboratory for Diagnosis of Zoonoses at the Institute of Microbiology and Immunology in Ljubljana, Slovenia. *I. ricinus* ticks were collected by flagging in TBE-endemic regions in Slovenia from 2005–2007 and were processed as described in [8]. Rodents were collected using live traps in years 2007–2008, from TBE-endemic regions in Slovenia, and were processed as described in [13]. Human serum samples were collected from year 2000–2010 as a part of routine diagnostic testing. Since the study was retrospective, informed consent from the patients was not obtained. Instead, the research was approved by the National Medical Ethics Committee of the Republic of Slovenia. Also, the principles of the Helsinki Declaration, the Oviedo Convention on Human Rights and Biomedicine, and the Slovene Code of Medical Deontology were followed in the conduct of this research. All human samples were anonymized and no additional sample was taken for the purpose of the study.

Samples were stored at −80°C until further processing. Sampling locations of tick and rodent samples as well as locations of residency of patients are shown in Table 1.

**Table 1:** TBEV sample information.

No.	SampleID	Host	Sampling/Residency location	Year	E protein gene sequence GenBank accession no. (PhC)	NS5 protein gene sequence GenBank accession no. (PhC)
1	H-47	human	Škofja Loka	2000	JQ654654 (A1)	ND
2	H-48	human	Kamnik	2002	JQ654655 (A1)	JQ654681 (A1)
3	H-49	human	Ribnica	2003	JQ654656 (A1)	JQ654685 (A4)
4	H-51	human	Draga-Crnomelj	2003	JQ654658 (A4)	JQ654686 (A4)
5	H-52	human	Nazarje-CE	2003	JQ654659 (A6)	JQ654682 (A6)
6	H-54	human	Trži 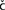	2006	JQ654661 (A1)	ND
7	H-55	human	Cerkno	2007	JQ654662 (A5)	JQ654683 (A5)
8	H-56	human	Ljubljana	2007	JQ654663 (A2)	JQ654684 (A2)
9	H-57	human	Trži 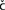	2009	JQ654664 (A1)	JQ654688 (A1)
10	H-58	human	Trži 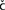	2009	JQ654665 (A3)	JQ654689 (A3)
11	H-59	human	Kranj	2009	JQ654666 (A1)	JQ654690 (A1)
12	H-60	human	Poljane nad Skofjo Loko	2010	JQ654667 (A3)	JQ654691 (A3)
13	H-61	human	Polhov Gradec	2009	JQ654668 (A1)	JQ654692 (A1)
14	H-62	human	Pivka	2009	JQ654657 (A2)	JQ654693 (A2)
15	H-63	human	Velenje	2009	ND	JQ654694 (A1)
16	H-64	human	Stara Cerkev	2010	JQ654660 (A4)	JQ654695 (A4)
17	H-66	human	Škofja Loka	2009	ND	JQ654697 (A1)
18	H-67	human	Ribnica	2010	ND	JQ654698 (A4)
19	H-68	human	Sodražica	2009	ND	JQ654699 (A2)
20	R-30	rodent	Rakovnik	2007	JQ654650 (A3)	JQ654678 (A3)
21	R-33	rodent	Tenetiše	2007	JQ654651 (A1)	ND
22	R-35	rodent	Rakovnik	2008	ND	JQ654679 (A3)
23	R-37	rodent	Rakovnik	2008	JQ654652 (A3)	JQ654680 (A3)
24	R-42	rodent	Vransko	2007	JQ654653 (A6)	ND
25	R-70	rodent	Rakovnik	2008	ND	JQ654700 (A3)
26	R-71	rodent	Rakovnik	2007	ND	JQ654669 (A3)
27	T-10	tick	Kamnik	2006	JQ654645 (A7)	JQ654673 (A7)
28	T-12	tick	Osolnik	2006	JQ654646 (A3)	JQ654674 (A3)
29	T-15	tick	Mozirje	2006	JQ654647 (A1)	JQ654675 (A1)
30	T-20	tick	Sodražica	2006	JQ654648 (A2)	ND
31	T-23	tick	Sodražica	2007	JQ654649 (A2)	JQ654676 (A2)
32	T-27	tick	Sodražica	2007	ND	JQ654677 (A2)
33	T-3	tick	Štefanja gora	2005	JQ654642 (A1)	JQ654670 (A1)
34	T-8	tick	Sodražica	2005	JQ654643 (A2)	JQ654671 (A2)
35	T-9	tick	Rakovnik	2006	JQ654644 (A3)	JQ654672 (A3)

ND = Not determined.

PhC = Phylogenetic cluster.

### Total RNA extraction and RT-PCR

Total RNA from human serum samples was extracted using Trizol® LS Reagent (Invitrogen Life Technologies™) according to the manufacturer's instructions. Total RNA from homogenized ticks or mice spleen was extracted using Trizol® Reagent (Invitrogen Life Technologies™) according to the manufacturer's instructions. RNA was stored at −20°C for up to a week or at −80°C for long term storage.

TBEV Real-Time RT-PCR positive samples were included in this study. Real-Time RT-PCR was performed as previously described in [20]. Partial TBE E gene was amplified and sequenced using primer pairs TBE1082 and TBEc2022 [21] or TBE ENV 3F (5′TGA GGG GAA GCC TTC AAT3′) and TBE ENV 3R (5′TCA TGT TCA GGC CCA ACC A3′). Partial TBE NS5 gene was amplified and sequenced using primer pairs TBE7547 and TBEc8732 [21]. RT-PCR was performed using the SuperScript® III One-Step RT-PCR System with Platinum® Taq High Fidelity (Invitrogen).

### Sequencing of PCR products

Amplified PCR products were purified with the Wizard® SV Gel and PCR Clean-Up System (Promega). Cycle sequencing was performed using the BigDye® Terminator 3.1 Cycle sequencing kit (Applied Biosystems). Sequencing reactions were purified with the BigDye® XTerminator™ Purification kit (Applied Biosystems) and analyzed with the 3500 Genetic Analyzer (Applied Biosystems).

### DNA sequence analysis

Nucleotide sequences were assembled and cropped to equal lengths using CLC Main Workbench software (CLC bio, Denmark) and were aligned in MEGA version 5 [22] using Muscle algorithm. For the complete genome analysis of the TBEV Ljubljana I strain we included in the alignment 52 complete genome sequences of TBEV representing all three TBEV subtype groups available. Complete genome sequence of OMSK hemorrhagic fever virus was used as an outgroup for the analysis. Accession numbers of all included sequences are presented in Figure 1. Nucleotide substitution model was selected based on Akaike's information criterion (AIC) in jModelTest, version 0.1.1 [23]. The general time-reversible model with gamma-distributed rate variation and a proportion of invariable sites (GTR+G+I) was employed for all phylogenetic analyses. Bayesian phylogenetic analyses were performed in MrBayes 3.2 [24] and Tracer version 1.5 [25]. Three independent MCMC runs of four chains each consisting of 10,000,000 generations were run to ensure effective sample sizes (ESS) of at least 200. Maximum clade credibility trees were depicted using FigTree version 1.3.1 [25].

### Recombination detection

TBEV E and NS5 protein gene sequences (757 bp and 933 bp in length respectively) which were obtained from the same samples were concatenated in CLC Main Workbench (CLC bio, Denmark). Concatenated sequences were aligned using Muscle algorithm in MEGA version 5 [22] and analyzed for putative recombination events using Rdp version 3.44 software (Heath L, et al 2006). An exploratory search was employed using methods RDP [26], GENECOV [27], Chimaera [28], MaxChi [29], BootScan [30], Siscan [31] and 3Seq [32].
